# Efficacy and safety of ‘dropless vitrectomy surgery’ and comparison of outcomes to standard of care topical therapy

**DOI:** 10.3389/fopht.2023.1215968

**Published:** 2023-07-28

**Authors:** K. V. Chalam, Harris Ahmed

**Affiliations:** Department of Ophthalmology, Loma Linda University Medical School, Los Angeles, CA, United States

**Keywords:** retinal detachment, topical drops, dropless vitrectomy, trimoxi, epiretinal membrane

## Abstract

**Objective/Background:**

To compare the effectiveness of intravitreal injection of triamcinolone acetonide/moxifloxacin (Tri-Moxi) with the standard eye drop regimen for controlling postoperative inflammation, intraocular pressure, infections, macular thickness, and visual acuity (VA) in patients undergoing pars plana vitrectomy for various retinal disorders.

**Subject/Methods:**

In this retrospective longitudinal study, patients who underwent vitrectomy using intravitreal Tri-Moxi at the end of surgery (Group 1) were compared with those who received standard topical steroid antibiotics (Group 2) in terms of intraocular inflammation, intraocular pressure, macular thickness based on optical coherence tomography, and visual acuity.

**Results:**

In total, 162 consecutive eyes (group 1 [81 eyes]; group 2 [82 eyes]) were included. VA improved by two lines in both groups at 90 days. In Group 1, preoperative VA (logMAR) was 0.92 (0.66) compared to 0.92 (0.75) in Group 2 (p = 1), while at 3 months, it was 0.61 (0.3) and 0.57 (0.3), respectively (p = 0.46). Group 1 showed superior outcomes concerning central foveal thickness. The average central foveal thickness CFT (µm) in group 2 preoperatively was 423 (95) and 348 (63) at 3 months compared to group 1 526 (109) and 306 (108) preoperatively and 3 months, respectively (p = 0.042). There was no statistically significant difference in the rate of elevated intraocular pressure between the two groups or anterior chamber cell reaction severity, and no cases of endophthalmitis were observed in either group.

**Conclusions:**

Tri-Moxi is effective after vitrectomy and is not inferior to standard postoperative topical therapy.

## Introduction

Vitreoretinal surgery is among the most common successful procedures in the world, with an anatomical success rate of over 90% (1). Advances in technology and techniques such as smaller gauge ports, self-sealing, and sutureless incisions have made this procedure safer, with successful outcomes in most cases. One of the most common concerns after vitrectomy is reducing postoperative inflammation and preventing microbial proliferation through anti-inflammatory and antibiotic agents. Macular edema and postoperative endophthalmitis are caused by inflammation and microbial invasion, respectively (2), which can lead to suboptimal anatomic and visual outcomes. Hence, appropriate measures should be taken to prevent such devastating complications.

Traditionally, topical eye drop therapy has been the mainstay for controlling inflammation and preventing infection in the postoperative period. Despite advances in many aspects of vitreoretinal surgery, the standard of care for eye drop routine has largely remained unchanged. Recently, the introduction of intravitreal triamcinolone acetonide/moxifloxacin (Tri-Moxi) has made cataract surgery a ‘dropless’ surgery option (3). In one study, subjects who received a transzonular injection of Tri-Moxi-Vanc showed rates of infection and inflammation similar to those with the standard prophylactic approach of topical medications (4). Another prospective study demonstrated similar outcomes for transzonular injection of Tri-Moxi-Vanc and a single drop compounded topical regimen and did not find a significant difference in macular thickness and change in intraocular pressure between the approaches (5). Bardoloi et al. found that most patients receiving a transzonular injection of Tri-Moxi had improved visual acuity and normal range intraocular pressure at 3 months follow-up (6).

As some cataract surgeons have begun moving away from the routine use of postoperative drops with good results, the clinical efficacy and approach may similarly succeed in vitreoretinal surgery. Topical therapy poses many challenges and adverse effects, including the burden of consistent drop usage, compliance issues, unpredictable dose delivery, and ocular surface toxicity. In addition to their expense, many patients lack the visual acuity and/or dexterity to place eye drops in their eyes reliably (7).

In this study, we compared the postoperative outcomes of intra-operative intravitreal injection of Tri-Moxi with those of the standard topical eye drop regimen after vitrectomy surgery. Our study findings demonstrate that Tri-Moxi injection at the end of vitrectomy effectively controlled postoperative inflammation, prevented infection, and was non-inferior to standard eye drop therapy. The results of our study aimed to optimize postoperative management after vitreoretinal surgery.

## Methods

### Study participants

This retrospective longitudinal cohort study was performed at the Loma Linda University Eye Institute, California, USA. Institutional Review Board approval was obtained; however, informed consent was not obtained, as this was a retrospective study. Electronic medical records of all patients who underwent vitrectomy by a vitreoretinal surgeon at the Loma Linda University Eye Institute from August 2019 to March 2021 were reviewed. The study groups included patients who received pars plana intravitreal injection of triamcinolone acetonide–moxifloxacin (Tri-Moxi; Imprimis Pharmaceuticals Inc., San Diego, CA, USA) (Group 1) and patients who received the standard steroid-antibiotic (Tobradex; tobramycin/dexamethasone, Novartis Pharmaceuticals Corporation, East Hanover, New Jersey, USA) eye drop taper (Group 2). The selection of intraoperative Tri-Moxi injection versus standard topical eye drop therapy was based on consecutive sampling. All patients had at least a 3-month postoperative follow-up period. Each subject underwent a complete ophthalmologic examination, visual acuity testing using the ETDRS charts, tonometry, anterior and posterior segment examination, and optical coherence tomography of the macula (Heidelberg Spectralis, Germany) at all pre- and postoperative visits. Eligible study subjects were patients at least 18 years of age, with no preoperative elevation in intraocular pressure or glaucoma, who had vitreoretinal pathology necessitating vitrectomy surgery and consistent follow-up at postoperative day 1, week 6, and day 90 visits.

### Procedure

Group 1 received pars plana intravitreal injection of triamcinolone acetonide-moxifloxacin at the end of the vitrectomy, and Group 2 received the standard steroid-antibiotic (Tobradex) eye drop only. Pars plana vitrectomy was performed using standard 23-gauge vitrectomy with a single 10-0 Nylon sutured incision site for the treatment of epiretinal membrane, vitreous hemorrhage, macular hole, rhegmatogenous and tractional retinal detachment repair, and silicone oil removal. Patients in Group 1 underwent pars plana intravitreal injection of triamcinolone acetonide–moxifloxacin (0.1 mL total of Tri-Moxi, for total drug delivery of 15 mg/mL of triamcinolone acetonide and 1 mg/mL of moxifloxacin) 4.0 mm posterior to the limbus in either the superotemporal or inferotemporal quadrant after completion of the case (Image 1). Medication was injected using a 30-gauge needle (4 mm length) on a tuberculin syringe. Group 2 received a steroid antibiotic (Tobradex) eye drop with instructions to administer one drop every four hours while awake for the first week with a subsequent taper over the next 5 weeks (6 weeks total).

### Assessment

Outcome assessments were performed via a chart review of the subjects’ medical records. The operative surgeon served as the evaluator at the post-operative visits. Demographic and clinical data were collected at baseline (preoperative) and 1 day, 6 weeks, and 3 months postoperatively. The assessments included distance visual acuity, intraocular pressure (IOP), standard anterior and posterior segment slit lamp examination, and indirect ophthalmoscopy fundus examination. The anterior chamber cell inflammation was graded from 0 to 4. IOP elevation was defined as an IOP ≥ 24 mmHg. OCT of the macula was performed for all patients preoperatively and at each postoperative visit. Postoperative complications were also recorded. (Image 2)

### Statistical analysis

Statistical analyses were performed using GraphPad software. The chi-square test was used to compare categorical variables. Statistical significance was set at P < 0.05.

## Results

Our study included 162 patients (81 in each group). Group 1 was the study group (**Table 1**) that received Tri-Moxi intravitreal injection, whereas Group 2 was the control group that received the standard eye drop regimen. All patients had complete follow-ups on days 1, 6, and 90. Efforts were made to minimize confounding by excluding patients with preoperative elevation in intraocular pressure or glaucoma and vitreoretinal pathology necessitating vitrectomy surgery. Patients were selected into groups 1 and 2 based on consecutive sampling, and systemic disease was not factored in.

**Figure 1:**
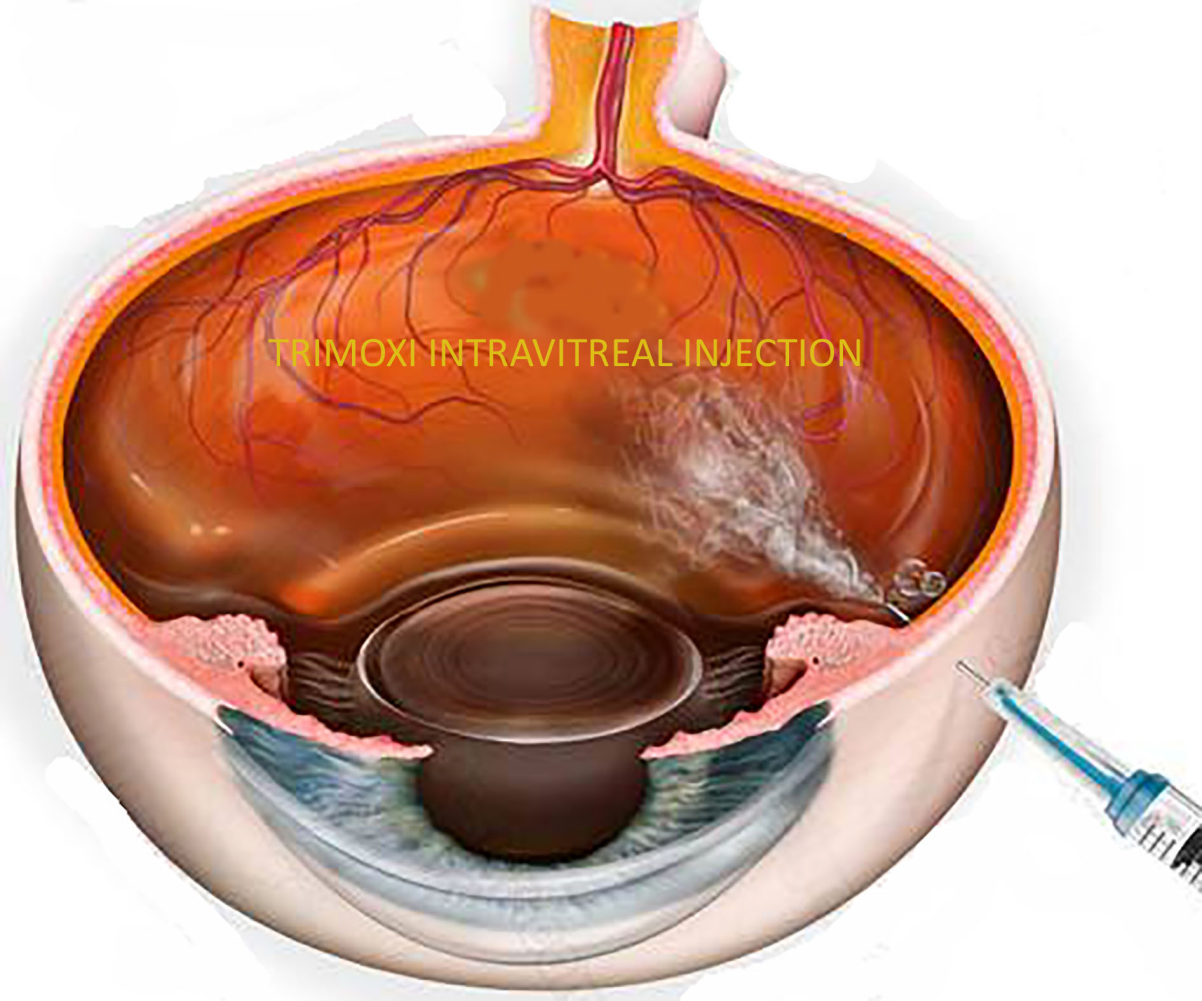
Illustration of Trimoxi intravitreal injection into inferior vitreous cavity at the end of pars plana vitrectomy.

The average age (**Table 1**) was 68.2 for Group 1 and 65.8 for Group 2 (p = 0.77). Group 1 included 43 females and 39 males, while Group 2 included 38 males and 42 females (p = 0.82, females = 0.86). Within Group 1, 39 patients were phakic; in Group 2, 41 were phakic (p = 0.86). 42 patients were pseudophakic in group 1 and 40 in group 2 (p = 0.99). Group 1 included 51 patients with an epiretinal membrane compared with 46 in Group 2, 11 with a non-clearing vitreous hemorrhage (secondary to proliferative diabetic retinopathy, retinal vein occlusion, and sickle cell retinopathy) compared with 12 in Group 2, 9 with a macular hole vs. 5 in Group 2, and 5 with silicone oil in the eye compared with 4 in Group 2 (**Figure 1**).

**Table 1:** Demographic Characteristics of Study Population.

	Group 1 (81 eyes)	Group 2 (81 eyes)
Age (y)	68.2	65.8
Gender		
*Male*	43	39
*Female*	38	42
Lens Status		
*Phakic*	39	41
*Pseudophakic*	42	40
Diagnosis		
*ERM*	51	46
*NCVH*	11	12
*MH Repair*	9	5
*RRD Repair*	5	14
*SIO Removal*	5	4

Group 1 – Control (Standard topical eye drop).

Group 2 – Tri-Moxi (Intravitreal injection).

ERM, Epiretinal Membrane; NCVH, Non-Clearing Vitreous Hemorrhage; RRD, Rhegmatogenous Retinal Detachment; MH, Macular Hole; SIO, Silicone oil.

**Figure 2:**
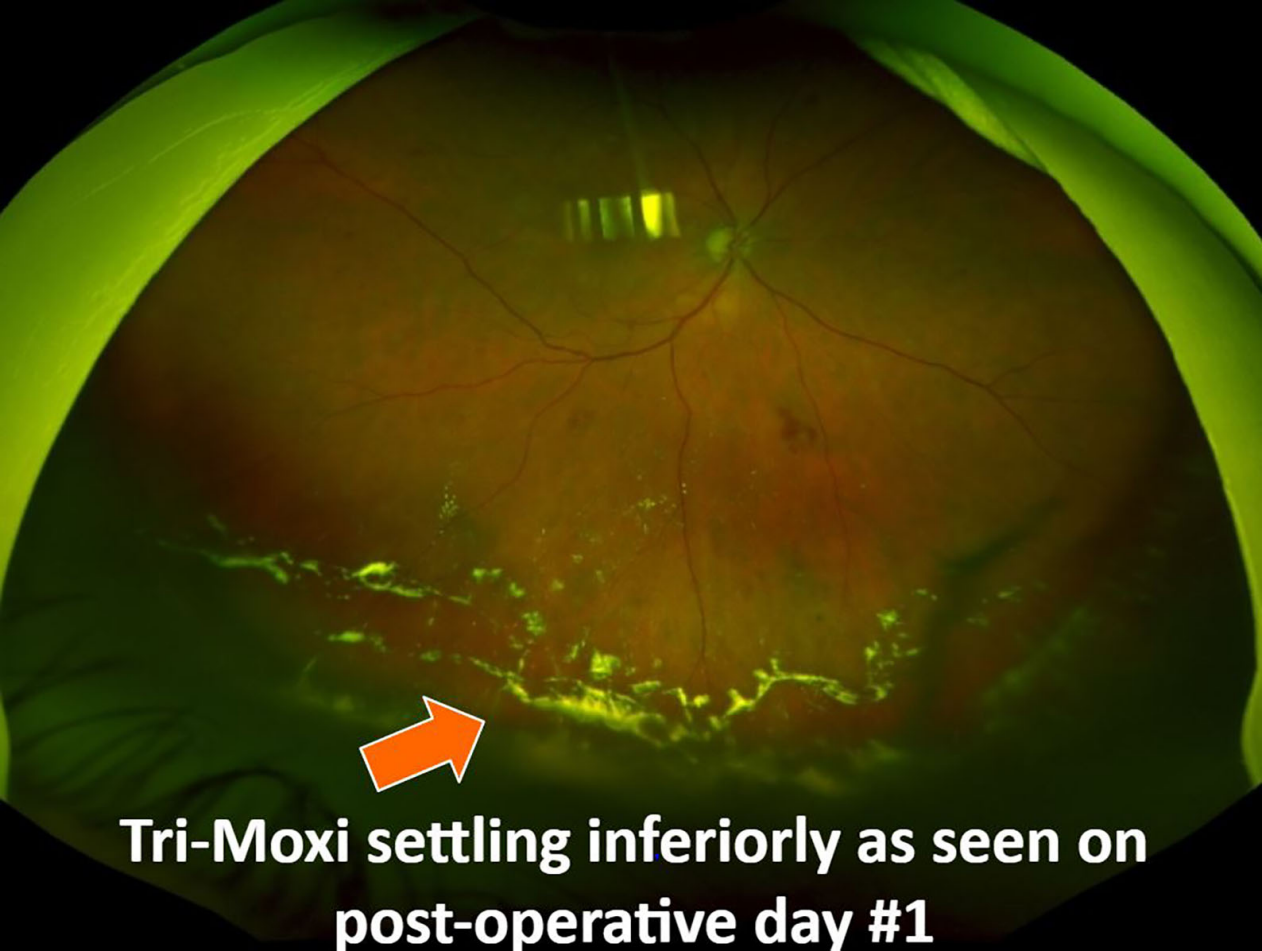
Fundus photograph showing Tri-Moxi deposition in the inferior vitreous cavity on post-operative day #1.

There was no statistically significant difference between the groups for most of the variables of interest, including the rate of infection, rise in postoperative intraocular pressure, and change in postoperative visual acuity (**Table 2**). The lack of statistically significant difference held across the underlying etiologies as well. The rate of infection was 0 in both groups. The mean IOP (mmHg) for Group 1 pre-operatively was 15.8 +/- 2.7 and 15.11 +/- 3.5 at 3 months post-op compared to Group 2, the IOP was 16.59 +/- 5.1 and 15.89 +/- 3.4 pre-operatively and at 3 months post-op, respectively. These differences were not statistically significant either preoperatively (p = 0.98) or 3 months postoperatively (p = 0.91). In terms of visual acuity (logMAR) in Group 1, preoperative visual acuity was 0.92 (0.66) compared to 0.92 (0.75]) in Group 2 (p = 1), while at 3 months, the visual acuity was 0.61 (0.3) and 0.57 (0.3), respectively (p = 0.46) (**Figure 2**).

**Table 2:** Description of primary and secondary outcomes of various parameters.

	Group 1 (81 eyes)	Group 2 (81 eyes)	P-Value
Rate of Infection (%)	0	0	
IOP (mmHg)			
*Pre-op*	15.8 ± 2.7	16.59 ± 5.1	0.98
*Post-op (3 mo)*	15.11 ± 3.5	15.89 ± 3.4	0.91
Visual Acuity (logMAR)			
*Pre-op*	0.92 (0.66)	0.92 (0.75)	
*Post-op (3 mo)*	0.61 (0.3)	0.57 (0.3)	0.46
OCT (CFT, um)			
*Pre-op*	423 (95)	526 (109)	
*Post-op (3 mo)*	348 (63)	306 (108)	
	0.001	0.001	0.042

Group 1 – Control (Standard topical eye drop), Group 2 – Tri-Moxi (Intravitreal injection).

OCT, Optical Coherence Tomography; CFT, Central Foveal Thickness.

Group 1 showed superior outcomes concerning the central foveal thickness on OCT. The mean average central foveal thickness (CFT, µm) in Group 2 preoperatively was 423 (95) and 348 (63) at 3 months postoperatively compared to 526 (109) and 306 (108) in Group 1 preoperatively and 3 months postoperatively, respectively. This difference in thickness between the groups at 3 months was statistically significant (p = 0.042) (**Figure 2**).

**Figure 3:**
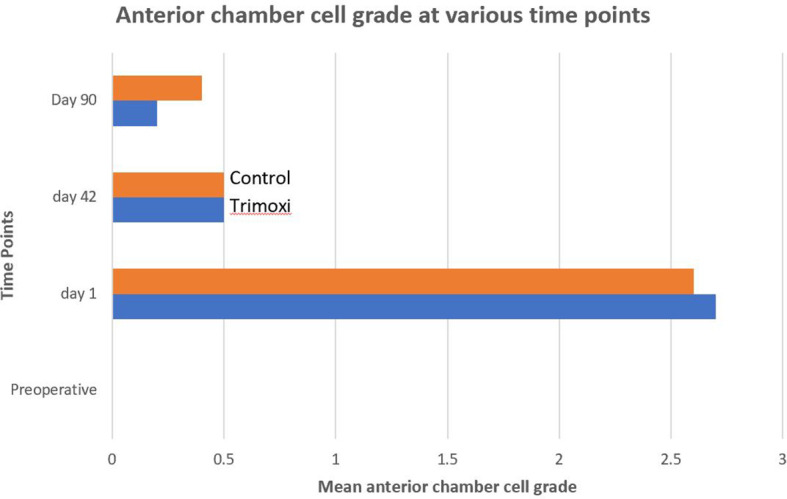
Comparison of Anterior Chamber Inflammation.

The postoperative anterior chamber cell reaction severity was lower by 35.0% and 35.7% at 1 day and 3 months after PPV in Group 1 compared with Group 2 (P =0.42). The intraocular inflammation severity was not statistically significantly different (**Figure 3**) between the two groups on postoperative day 90 (P = .57).

## Discussion

Control of intraocular inflammation, normal postoperative intraocular pressure, and infection prevention are common goals of postoperative care after vitreoretinal surgery. To our knowledge, our study is the first to compare the effect of Tri-moxi intravitreal injection with standard topical eye drop usage after vitreoretinal surgery.

These results indicate that Tri-Moxi intravitreal injection is an effective method for controlling intraocular inflammation after vitrectomy surgery. Postoperative inflammation decreased quicker in the Tri-Moxi group than in the standard topical eye drop therapy group, without a statistically significant difference in intraocular pressure at all visits between the two groups. Additionally, no secondary postoperative IOP elevation was observed in either group. It is possible that no pressure spike was noted in the study group due to using a 15 mg/ml dosing as opposed to the traditional 40 mg/ml intravitreal triamcinolone dosage. None of the patients developed postoperative infections. Overall, there was no statistically significant difference between the two groups outside of the change in central foveal thickness, which was more favorable in patients in Group 1. This difference may be due to the long-acting nature of intravitreal steroids.

The data regarding using an intravitreal ‘dropless’ regimen in vitreoretinal surgery remains limited. Secondary ocular hypertension is a commonly recognized complication of intravitreal steroid use. It is well known that topical steroids can induce an elevation in IOP; however, drops can easily be discontinued, whereas intravitreal steroid agents would require vitrectomy to remove the offending agent. Our study found no difference between the Tri-Moxi and standard topical eye drop regimen groups regarding elevated postoperative IOP. None of the patients required IOP-lowering therapy for elevated IOP postoperatively. In a meta-analysis by Jonas et al., IOP readings >21 mmHg were measured in 41.2% of patients after intravitreal injection of 20 mg triamcinolone acetonide (8). However, studies have shown that the injection of low-dose triamcinolone acetonide up to 3 mg has a lower incidence of IOP spikes than those seen with postoperative eye drops (9). In line with our study, Nassiri et al. did not find a difference in IOP between Tri-Moxi and standard topical therapy groups after cataract surgery (10). Various topical steroid agents may also cause steroid responses at varying rates depending on the specific steroid agent used. Although a significantly higher mean IOP has been observed with topical dexamethasone use, Laurell et al. reported that no patient exhibited an increase in IOP of >10 mmHg (11). In another meta-analysis by Kiddee et al., 32% of individuals developed secondary ocular hypertension following 4 mg intravitreal triamcinolone use (12).

Other potential concerns regarding the use of an intravitreal dropless’ regimen in vitreoretinal surgery include compounding errors, unclear pharmacokinetics of the intravitreal agent, development of antimicrobial resistance, steroid-induced ocular hypertension, technical or mechanistic issues associated with intravitreal injections, and postoperative foggy vision and floaters (13). There were reports of temporary floaters in our patient population attributable to Tri-Moxi injection; however, it did not affect long-term visual outcomes. None of the patients with floaters had vitreous cells or signs of sterile inflammation. All injections were performed using the pars plana approach without any complications. We found that injection of Tri-Moxi via the inferotemporal quadrant allowed for better visibility of the macula on postoperative day 1 compared to those who received the injection via the superotemporal quadrant. We also found the utility of a 4 mm length 30-gauge needle to be beneficial in allowing for proper evaluation of the macula and posterior pole on the day after surgery. Although concerns exist regarding the possible risk of retinal tear or detachment with the addition of intravitreal injection, we did not observe any such complications in our study.

Intravitreal delivery of antibiotic-steroid combination medications is an alternative to the standard eye drop regimen for postoperative care in ocular surgery. Other alternatives include subconjunctival drug delivery. Atchison et al.’s retrospective consecutive case series found that a single subconjunctival 4 mg triamcinolone acetonide injection at the end of vitreoretinal surgery may represent a reasonable alternative to steroid eye drop taper (14). Reports in the literature have found that the relative effectiveness of Tri-Moxi-Vanc injected transzonularly into the vitreous had similar outcomes compared to the standard prophylactic approach of topical therapy, including similar rates of infection and inflammation with greater convenience and no apparent difference in therapeutic effect (4, 5). Another recent retrospective study by Garcia-O’Farrill et al. used a subconjunctival injection of cefazolin-dexamethasone and sub-Tenon’s Kenalog at the time of 25- or 27-gauge vitrectomy surgery in 89 eyes and found no cases of postoperative endophthalmitis, also suggesting an alternative to topical eye drops (15). Compared to the subconjunctival injection of a drug, posterior segment drug delivery methods through a pars plana approach directly provide sustained drug transfer to the surgery site. However, these studies evaluated the outcomes after cataract surgery.

Reports in the literature regarding the incidence of postoperative infectious endophthalmitis following pars plana vitrectomy remain low (0.02-0.05%) compared to other intraocular surgeries (16, 17). The intraoperative use of steroids was previously controversial as a potential risk factor for developing a postoperative infection. In a retrospective multicenter study of 1,886 eyes that underwent triamcinolone-assisted vitrectomy, only one patient (0.05%) had acute endophthalmitis (18). In our study, there were no cases of endophthalmitis in either study group; however, a larger sample size is warranted for a definitive conclusion regarding the rate of endophthalmitis.

Previous studies have consistently found a significant correlation between visual acuity and central macular thickness (18). Postoperative control of intraocular inflammation is vital for preventing cystoid macular edema (CME) and obtaining good visual outcomes. The incidence of post-vitrectomy macular edema ranges from 5-47%; however, it depends, in part, on pre-existing retinal conditions (19, 20). Prophylactic treatment with CME is geared towards therapeutic targets against these inflammatory mediators. Regarding control of post-operative inflammation and edema, underlying etiology contributes to outcomes. In our study, however, subgroups did not differ significantly concerning the postoperative inflammatory course.

**Figure 4:**
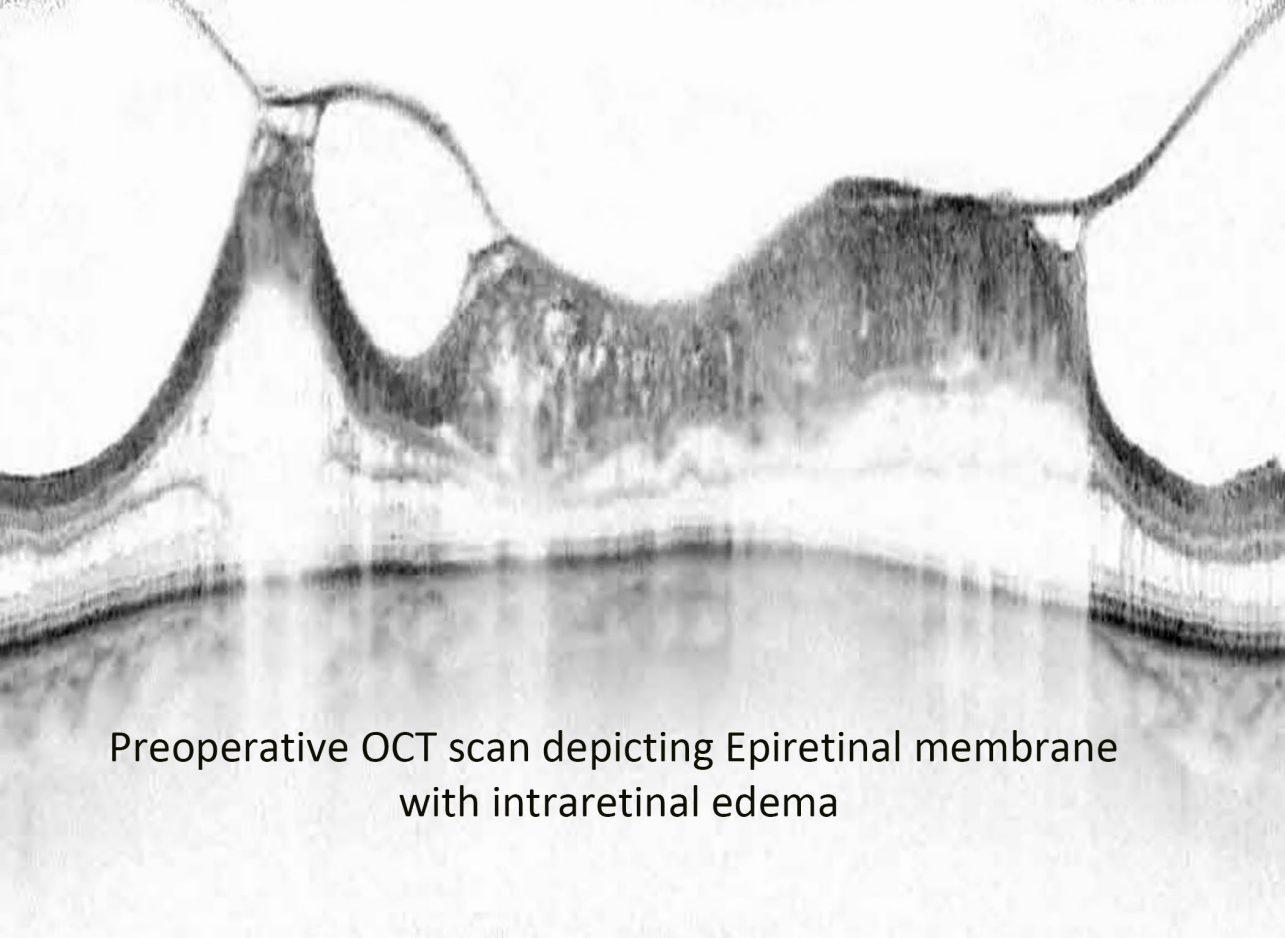
Preoperative OCT image of an epiretinal membrane associated with severe intraretinal edema.

**Figure 5:**
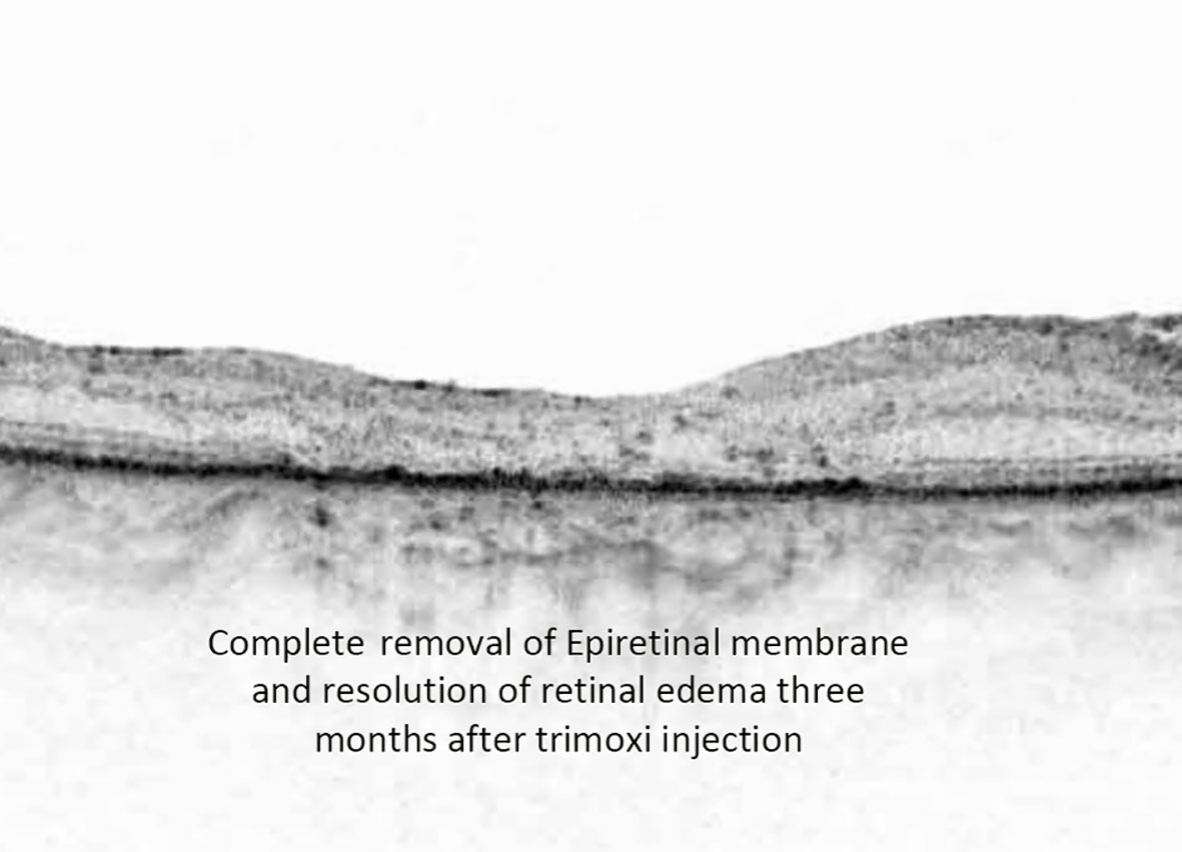
Postoperative OCT image depicting complete removal of epiretinal membrane along with the resolution of retinal edema at three-month follow-up time point.

Alam et al. showed that the incidence of CME after vitrectomy and epiretinal membrane peel was 12.8%, while Merad et al. found the incidence was 28% after rhegmatogenous retinal detachment repair (21, 22). They also showed that the injection of intravitreal triamcinolone acetonide resulted in a significant decrease in CME and improved vision after 1 month. In our study, there was a statistically significant difference in the central foveal thickness (CFT) on optical coherence tomography (OCT) 3 months post-vitrectomy between the triamcinolone acetonide-moxifloxacin group (CFT 306 µm) and the standard eye drop regimen group (348 µm) (**Figures 4**, **5**). There was a difference in visual acuity at the 3-month postoperative visit between the two groups (VA 20/70 in group 1 and 20/100 in group 2; however, this difference was not statistically significant.

The current study had some limitations. The assessment of cellular reactions in the anterior chamber helps evaluate the severity of inflammation postoperatively. Although our grading system was based on the assessment by one provider, grading of the cells in the anterior chamber during slit-lamp examination is subjective and prone to intra-observer variability and measurement error. A laser flare cell meter may provide a noninvasive *in vivo* imaging and quantification tool by objectively measuring scattered light (22). In addition, we included patients who had undergone vitreoretinal surgery for all types of retinal disorders. A study stratifying the postoperative results of Tri-Moxi in specific retinal conditions with a larger patient population may better control for possible confounders and is suggested for future analyses. It is also unknown if patients were actually compliant with their postoperative eye drop regimen, which could skew the results of our study. Another limitation is that our current analysis is limited to data until the postoperative month 3 visit. Longer-term follow-up data is the focus of another analysis. Lastly, this was a single surgeon study, and the operative surgeon knew which patients received intravitreal Tri-Moxi versus standard drop therapy.

The advent of ‘dropless’ surgery is on the rise as a more practical technique for intra-operative delivery of a combination of an antibiotic and steroid. This may eventually completely mitigate the need for postoperative topical medication use. In theory, the concept behind the use of Tri-Moxi is attractive to both physicians and patients. Postoperative drops may be toxic to the corneal epithelium, resulting in ocular surface irritation and blurred vision. Associated expenses, application techniques, and potential patient noncompliance with topical drops for many weeks postoperatively can burden patients and physicians.

In conclusion, intravitreal injection of triamcinolone acetonide-moxifloxacin during vitreoretinal surgery appears to be non-inferior to standard topical eye drop therapy in terms of post-operative inflammation control, the rate of secondary ocular hypertension, improvement in central macular thickness, and prevention of postoperative infection. Therefore, Tri-Moxi injections can be considered a favorable substitute for standard topical therapy, modernizing postoperative care and improving outcomes and patient satisfaction.

## Data availability statement

The raw data supporting the conclusions of this article will be made available by the authors, without undue reservation.

## Ethics statement

The studies involving human participants were reviewed and approved by Loma Linda University IRB. Written informed consent from the participants’ legal guardian/next of kin was not required to participate in this study in accordance with the national legislation and the institutional requirements.

## Author contributions

KC designed the study, analyzed the data, and wrote the manuscript. HA collected the data and prepared the figures and tables. All authors contributed to the article and approved the submitted version.
